# Stimulated Whole Blood Cytokine Release as a Biomarker of Immunosuppression in the Critically Ill: The Need for a Standardized Methodology

**DOI:** 10.1097/SHK.0000000000000557

**Published:** 2016-04-15

**Authors:** Elisabetta Segre, James N. Fullerton

**Affiliations:** Centre for Clinical Pharmacology, Division of Medicine, University College London, London, UK

**Keywords:** Diagnostic, endotoxin tolerance, immunoadjuvant, lipopolysaccharide, sepsis, stratified medicine, therapeutics, tumor necrosis factor α

## Abstract

**Objective:**

Reduced *ex vivo* lipopolysaccharide (LPS) stimulated whole blood pro-inflammatory cytokine release is a hallmark of immunosuppression in the critically ill and predicts adverse clinical outcomes. No standard technique for performing the assay currently exists. The impact of methodological heterogeneity was determined.

**Design, Setting, Subjects, and Interventions:**

Clinical experimental study set in a research laboratory. Venous blood from 5 to 10 healthy volunteers/experiment (total participant group: 18 subjects, 72% men, mean age 32) was stimulated *ex vivo* to evaluate the effect of variables identified via literature review on tumor necrosis factor-α (TNFα) release. These included sample handling, stimulation technique, and incubation conditions. Reporting convention was additionally assessed.

**Main Results:**

Measured TNFα release was significantly altered by source of LPS, concentration of LPS employed, duration and temperature of incubation prior to supernatant aspiration, and predilution of blood (repeated measures ANOVA, all *P* < 0.01). Sample handling prior to stimulation (anticoagulant employed, time to LPS addition, and storage temperature) also caused significant alterations in TNFα release. Considerable interindividual variation was observed (range 1,024–4,649 pg/mL, mean 2,339 pg/mL). Normalization by monocyte count and pretreatment with a cyclooxygenase inhibitor (indomethacin 10 μM) reduced the coefficient of variation from 47.17% to 32.09%.

**Conclusions:**

Inconsistency in interlaboratory methodology and reporting impairs interpretation, comparability, and reproducibility of the *ex vivo* LPS-stimulated whole blood cytokine release assay. A standardized validated technique is required. The advent of trials of immunoadjuvant agents renders this a clinical imperative.

## INTRODUCTION

Late mortality in critically ill patients with severe sepsis, or following trauma, burn or major surgery, is now thought due to the predominance of anti-inflammatory processes. Whether excessive, sustained, or secondary to a failure of the immune system to recover from their functional consequences, the net effect is one of immunosuppression characterized by susceptibility to opportunistic infection and acquisition of secondary sepsis (1,2). Therapeutically, this may be addressed via augmentation of the immune system with immunoadjuvant agents (3), an approach that has demonstrated initial promise in selected septic patients (4,5).

The key to an immunorestorative strategy is patient stratification—identifying individuals with clinically meaningful immunosuppression who may benefit from treatment. To date, two interrelated biomarkers have repeatedly proven predictive of acquisition of both nosocomial infection and mortality and have consequently been used to guide inclusion in clinical trials: monocyte human leukocyte antigen DR (mHLA-DR) expression (6,7) and *ex vivo* lipopolysaccharide (LPS)-stimulated whole blood (WB) tumor necrosis factor α (TNFα) release (8–10). While a standardized methodology and reporting tradition has been established for mHLA-DR (11), the same is not true for *ex vivo* cytokine release, potentially leading to disparities in case definition, identification, treatment allocation, and outcome determination that impair data interpretation and comparability.

This study sought to identify key inter-laboratory differences in sample handling, technical performance of the WB *ex vivo* cytokine release assay, and result reporting convention, determining their impact on measured TNFα and hence quantification of immune competence.

## MATERIALS AND METHOD

### Study design, setting, and subjects

Eighteen healthy volunteers (13 men [72.2%], mean age 32 years) were recruited, 5 to 10 individuals participating in each experiment dependent on the variable evaluated. All samples were obtained and processed in a clinical research laboratory. Ethical approval for the protocol was provided by the University College London Research Ethics Committee (project ID: 4332/001).

### Data collection

Health Volunteer Blood: Venous blood was collected and stored in lithium heparin (LH, Grenier Bio-One Vacuette), ethylenediaminetetraacetic acid (EDTA), or sodium citrate (Na Cit, both BD Biosciences Vacutainer) containing tubes prior to stimulation.

Stimulation: Unless otherwise stated 1 mL heparinized WB was diluted 1:5 in RPMI (Gibco, Grand Island, NY) in 15 mL polypropylene tubes (Fisher Scientific, Grand Island, NY) and stimulated for 6 h (37°C, 250 rpm) with 1 ng/mL LPS (*Salmonella abortus equi* S-form [TLR*grade*], Enzo Life Sciences, Farmingdale, NY), after Kox et al. (12). Blood was stimulated within 30 min of draw. After incubation samples were centrifuged at 2000 g for 10 min at 20°C and supernatant stored at −80°C. Three technical repeats (tubes/individual/condition or time-point) were performed.

Variables: On the basis of the results of the literature review the core technique was modified to evaluate the effect of LPS type (*Escherichia coli 0111:B4* and *055:B5* [Sigma-Aldrich, St. Louis, Mo], *Salmonella abortus equi*, *Salmonella Minnesota* R-form serotype: R595 [Hycult]), LPS concentration (1 pg/mL to 100 ng/mL *Salmonella abortus equi*), duration of stimulation (30 min, 1, 2, 4, 6, 8, 24 h), anticoagulant employed (LH, EDTA, Na Cit), incubation conditions (temperature [20°C vs. 37°C], agitation [0 rpm vs. 250 rpm]), sample dilution (none, 1:1, 1:5, 1:10), and sample handling (time to stimulation [2, 4, 6, 12 h], storage temperature [4°C, 20°C, 37°C]). In addition, the effect of pretreating the blood with a non-steroidal anti-inflammatory drug (indomethacin 10 μM [Sigma-Aldrich], a dual cyclooxygenase 1 and 2 inhibitor) was evaluated. Where not supplied in solution LPS was reconstituted in sterile deionized water and vortexed extensively prior to use.

TNFα and Monocyte Quantification: The concentration of TNFα was determined via enzyme-linked immunosorbent assay according to the manufacturer's instructions (Duoset, R&D systems, Minneapolis, MN). Differential leucocyte counts were performed via a Sysmex XE2100 flow cytometric analyzer by The Doctors Laboratory (London, UK).

### Statistical analysis

Data points represent the average of three technical repeats, each assayed in duplicate. The D’Agostino–Pearson test was used to test for normality. Differences between categorical variables were tested for using either repeated measures one-way or two-way analysis of variance (RM-ANOVA, incorporating the Greenhouse–Geisser correction) with Tukey multiple comparisons test, or paired *t* tests. Statistical significance was set at *P* < 0.05. All analyses were conducted in Prism 6 (GraphPad Software).

## RESULTS

**Fig. 1:**
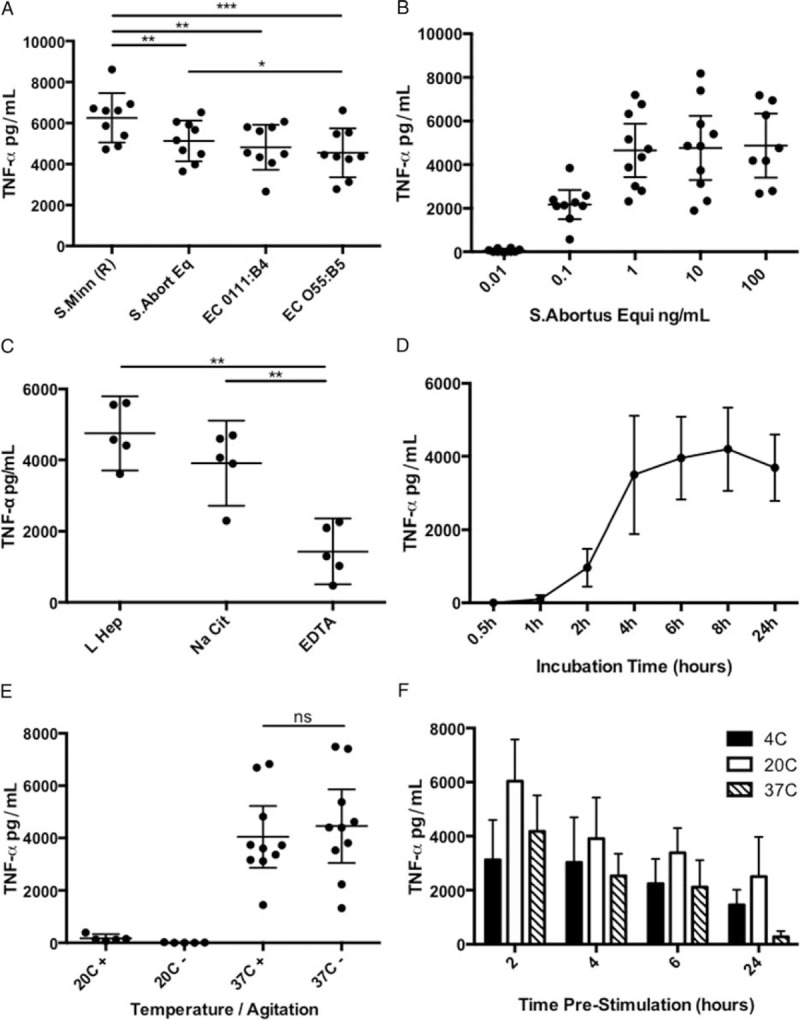
Effect of discrete variables on the *ex vivo* lipopolysaccharide (LPS)-stimulated whole blood (WB) cytokine release assay.

Stimulation with LPS (1 ng/mL) from different bacterial sources elicited significantly different concentrations of TNFα (*P* < 0.001, one-way RM-ANOVA, Fig. 1A), with “rough”-LPS derived from *S. minnesota* causing significantly greater TNFα release than the 3 “smooth”-LPS sources (all *P* < 0.01, Tukey). Cytokine release was further altered by the use of alternate anticoagulants (*P* < 0.001, one-way RM-ANOVA) being significantly lower when EDTA was employed than either LH or Na Cit (both *P* < 0.01, Tukey, Fig. 1C). While incubation of stimulated WB at 37°C was necessary—samples at 20°C releasing minimal TNFα (mean 89.27 pg/mL, Fig. 1E) —no significant difference was observed between agitated (mean 4,049 pg/mL, SD 1653) and nonagitated samples (4,459 pg/mL, SD 1967; *P* = 0.15, paired *t* test). Stimulation with either increasing doses of LPS (*S. abortus equi*), or for variable periods of time, predictably led to differential concentrations of TNFα in the supernatant when aspirated; 1 ng/mL and incubation time >4 h leading to maximal or equivalent responses (Fig. 1B and D). Spontaneous production of TNFα in un-stimulated blood was not observed.

WB (1 mL) from healthy volunteers was stimulated in triplicate with (A) LPS (1 ng/mL) from four different bacterial sources (*Escherichia coli 0111:B4* and *055:B5*, *Salmonella abortus equi* S-form [SAE], *Salmonella Minnesota* R-form serotype: R595). TNFα from un-stimulated cells was undetectable. B, n = 9. Different concentrations of LPS (*SAE*, 10 pg/mL to 100 ng/mL). C, n = 10. Alternate anticoagulants: lithium heparin, sodium citrate or EDTA (all SAE, 1 ng/mL). D, n = 5. For different lengths of time prior to supernatant removal (SAE, 1 ng/mL, 30 min to 24 h). E, n = 5. In alternate incubation conditions either 20°C or 37°C, with (+) or without (−) agitation. F, n = 10. Variations in sample handling (time to stimulation [2, 4, 6, 12 h] and storage temperature [4°C, 20°C, 37°C]). n = 5 per condition. Lines denote mean and 95% confidence intervals. Differences between categorical variables were tested for using either repeated measures one-way or two-way analysis of variance with Tukey multiple comparisons test, or paired *t* tests. Statistical significance is indicated as ^∗^*P* < 0.05, ^∗∗^*P* < 0.01, ^∗∗∗^*P* < 0.001. EDTA indicates ethylenediaminetetraacetic acid; ns, non-significant; TNFα, tumor necrosis factor α.

**Fig. 2:**
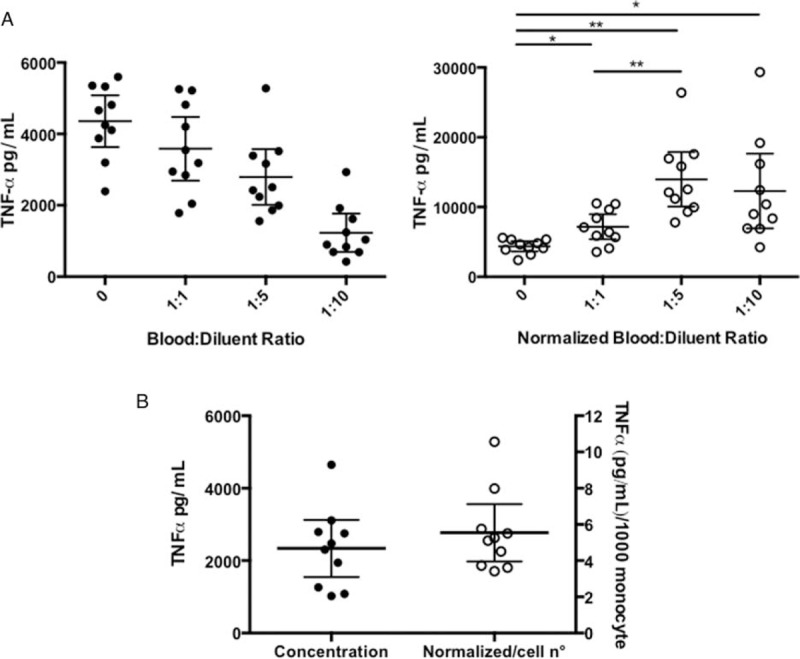
Variations in reporting convention of data from the *ex vivo* LPS-stimulated WB cytokine release assay.

Variations in sample handling prior to LPS stimulation caused discrepancies in assayed TNFα. Both time to LPS addition and storage temperature significantly effected supernatant TNFα concentration (both *P* < 0.001, 2-way RM ANOVA), increasing time leading to lower release and 20°C storage eliciting significantly greater release than either 4°C or 37°C (both *P* < 0.001, Tukey). No significant interaction was observed between the two variables (*P* = 0.2). Predilution of blood, for ease of technical performance and to enhance supernatant yield, as expected, leads to a fall in TNFα concentration per unit supernatant with an increased ratio of media to WB (Fig. 2A). This decrease was however not directly proportional, normalization by dilution factor failing to create equivalence between technical approaches (*P* < 0.01, one-way RM-ANOVA).

A, Left panel—WB (1 mL) was stimulated (*SAE*, 1 ng/mL) either un-diluted or prediluted with RPMI at a 1:1, 1:5, or 1:10 ratio. n = 10 per condition. Right panel—measured concentrations were normalized by dilution factor to examine for equivalence. Lines denote mean and 95% confidence intervals. Differences were tested for using repeated measures one-way analysis of variance with Tukey multiple comparisons test. Statistical significance is indicated as ^∗^*P* < 0.05, ^∗∗^*P* < 0.01. B, TNFα expressed as either an absolute concentration in aspirated supernatant or as a factor of cell number (/1000 monocytes). n = 10. TNFα indicates tumor necrosis factor α; WB, whole blood.

Considerable interindividual variation in LPS-stimulated TNFα release was observed, the supernatant of heparinized blood from 10 volunteers stimulated with 1 ng/mL *S. abortus equi* (6 h, 1:5 dilution, 37°C, agitated) having measured concentrations ranging from 1,024 to 4,649 pg/mL (mean 2,339 pg/mL, Fig. 2B). Expression of assayed TNFα as a function of cell number (pg/mL per 1,000 monocytes) rather than as an absolute concentration in supernatant (pg/mL) resulted in a decrease in the co-efficient of variation (39.95% vs. 47.17% respectively, Fig. 2B). The addition of a cyclooxygenase inhibitor to the assay both decreased the co-efficient of variation (37.67% vs. 47.17%) and significantly increased the measured TNFα concentration (panel A, Supplemental Figure 1, Supplemental Digital Content 1, LPS alone mean 2339 pg/mL, NSAID LPS 3108 pg/mL, *P* < 0.001, RM *t* test). Normalization of these values by monocyte count further reduced the co-efficient of variation (32.09%, Panel B, Supplemental Figure 1, Supplemental Digital Content 1). While intra-individual variation in serially assessed TNFα release was additionally noted, the statistical significance of this was not tested (panel C, Supplemental Figure 1, Supplemental Digital Content 1).

## DISCUSSION

*Ex vivo* LPS-stimulated WB TNFα release is a biomarker of monocyte function and reflective of global immune competence, persistent reduction being predictive of adverse outcomes, and its restoration an indicator of clinical improvement (4,9,10,13). However, to date, laboratories have employed divergent techniques to determine and report this key metric (see Table 1) (14–18), impairing interpretation, comparability, and reproducibility. No clear rationale underlies these alternate approaches.

Selection of LPS source, LPS concentration, duration and temperature of incubation, anticoagulant and decision to dilute the blood varied extensively between laboratories, and were all demonstrated to significantly alter the assay result. Sample handling prior to stimulation, rarely reported in manuscripts, was additionally observed to contribute variance—both time to LPS addition and WB storage temperature— impacting subsequent TNFα release. Reporting inconsistencies further obfuscate results, studies presenting either concentration of TNFα alone (4) or expressing this as a factor of cell number (total WBC count (19), monocyte count (12)), and failing to describe the number of biological and/or technical repeats undertaken per time-point/condition per individual. Both normalizing TNFα concentration to monocyte count and pretreatment of the blood with a cyclooxygenase inhibitor were found to synergistically reduce the coefficient of variation; however, the functional and diagnostic relevance of this is unclear.

These results are not unexpected. It has long been known that varying the LPS dose or duration of incubation alters the readout of the *ex vivo* WB assay (14,15). Nor is it surprising that sample handling, different sources of LPS, dilution of the blood, or choice of anticoagulant alters measured TNFα concentrations. What is remarkable however is the lack of derivation and adoption of a uniform method and reporting standard for this important test of immune dysfunction: a deficiency that needs to be addressed. Stratified immunoadjuvant therapy holds great promise for critically ill patients (3,20). Establishing a panel of biomarkers with associated clinically relevant definitions and predictive value, to determine who to treat, when to treat them, and what to administer will be critical to its success (21). It is vital that avoidable methodological heterogeneity does not confound inclusion criteria, determination of outcome, and thus the external validity of future clinical trials.

It should be noted that this study was not designed to define a “gold-standard” method of undertaking the *ex vivo* WB stimulation assay, instead seeking to identify key sources of methodological variance and highlight their implications. Participants were healthy volunteers, a “core” method was selected and only one method of TNFα measurement was performed. Response variation may have differed had an alternate system and population been employed. In particular, the importance of normalizing TNFα release to monocyte count—more variable in acute illness—to accurately gauge immune competence may have been underestimated. The blood collection tubes employed were sterile and from one batch, yet not guaranteed to be pyrogen free. Additional technical factors not elaborated here may also impact measured TNFα release including the method of LPS extraction, purification and solubilization, and the testing of either fresh or frozen supernatants. Future work may address these deficiencies and should seek to formulate and validate the predictive and diagnostic value of a standardized assay—in conjunction with additional metrics of immunosuppression—as a clinical imperative.

## Supplementary Material

Supplemental Digital Content

## Figures and Tables

**Table 1:** Heterogeneity in methodology employed to perform the *ex vivo* lipopolysaccharide (LPS)-stimulated whole blood cytokine release assay

	Ertel 1995 (14)	Ogata 2000 (16)	Wang 2000 (15)	Heagy 2003 (17)	Ploder 2006 (9)	Allen 2006 (18)	Hall 2011 (10)	Kox 2011 (12)
LPS type	*E. Coli 055:B5*	*E. Coli 127:B8*	*E. Coli 026:B7*	*E. Coli 0111:B4*	*S. Abortus Equi*	*N. Meninigitides*	*S. Abortus Equi*	*E. Coli O55:B5*
LPS concentration	1 ng to 1 μg/mL	10 ng/mL	10 ng/mL	10 ng/mL	500 pg/mL	10 μg/mL	500 pg/mL	1 ng/mL
Diluent type	None	Saline	None	RPMI	RPMI	RPMI	NI	RPMI
Blood /diluent (ratio)	5 mL/0 (N/A)	NI/5x (1:5)	NI/0 (N/A)	1–3 mL/0 (N/A)	50 μL/0 (N/A)	500 μL/500 μL (1:1)	50 μL/500 μL (1:10)	1 mL/4mL (1:5)
Incubation/agitation	1–24 h/yes	4 h/NI	2–24 h/yes	3 h/yes	4 h/NI	24 h/NI	4 h/NI	24 h/NI
Anticoagulant	Heparin	Heparin	Citrate	Heparin	Heparin	Heparin	Heparin	Heparin
Sample handling (time/temp.)	NI/NI	NI/NI	NI/NI	NI/NI	NI/Ice	NI/NI	1 h/NI	NI/NI
TNFα quantification	Bioassay	NI	EIA and bioassay	ELISA	Chemi-luminescence	NI	Chemi-luminescence	Luminex Assay
Results expression	U/mL/1×10^6^ monocytes	U/mL	pg/mL	pg/mL	ng/mL	pg/mL	pg/mL	pg/1000 monocytes
Indicative results	535.9 ± 75	240 ± 36	1,449–2,484	6,706 ± 715	1.05–2.8	900 ± 50	900–2,172	55 ± 5

Eight published papers (first author, year of publication) were selected to exemplify diversity in technical performance of the assay along key variables (LPS type and concentration, use of diluent and ratio to blood, incubation period after stimulation [all at 37°C], use of agitation during this period, choice of anticoagulant), sample handling prior to analysis (time and storage temperature from collection to stimulation) method of tumor necrosis factor α (TNFα) measurement and reporting of results (absolute TNFα concentration in supernatant or whether normalized to cell number). The range, mean or median (±SEM or IQR respectively) of TNFα concentrations reported in the control group in each study is also displayed. ELISA indicates enzyme-linked immunosorbent assay; N/A indicates not applicable; NI, not indicated.
