# Supplementary figures and images for: Stimulated Whole Blood Cytokine Release as a Biomarker of Immunosuppression in the Critically Ill: The Need for a Standardized Methodology
Source: Shock. 2016 Apr 15;45(5):490–4. doi: 10.1097/SHK.0000000000000557 (PMC4836558; doi:10.1097/SHK.0000000000000557)

**A**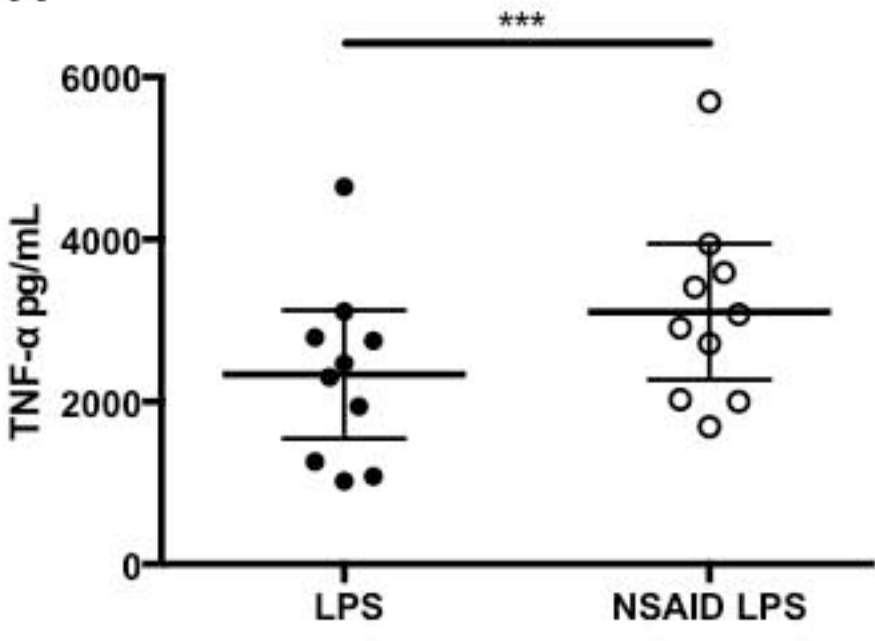**B**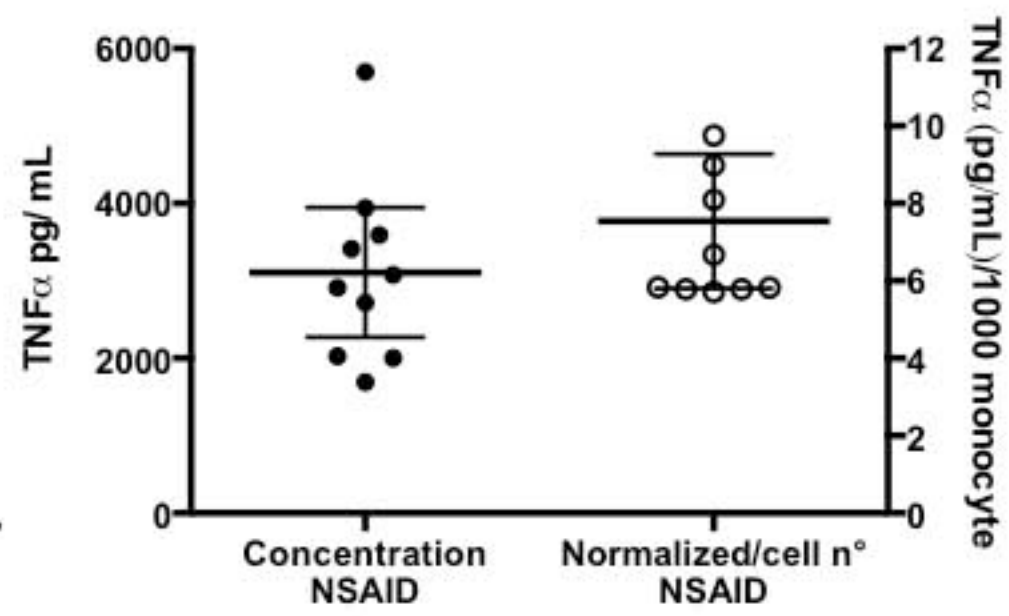**C**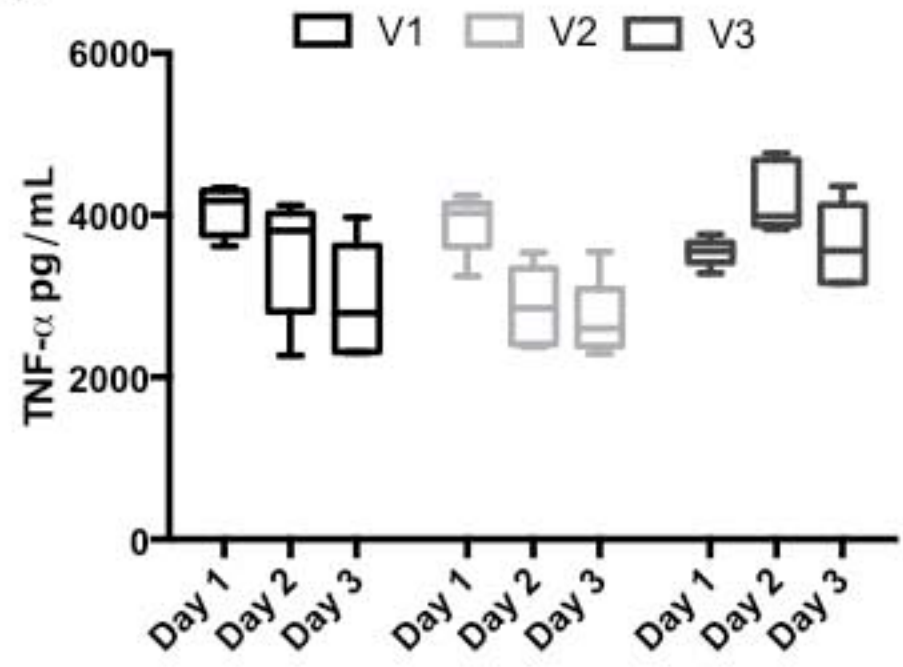

Supplement: Supplemental Digital Content [file shk-45-490-s001.pdf]
